# The Evolution of a Casualty Clearing Station on the Western Front

**Published:** 1937

**Authors:** Richard C. Clarke

**Affiliations:** Physician to the Bristol Royal Infirmary


### The Bristol Medico-Chirurgical Journal

"*Scire est nescire, nisi id me*
*Scire alius sciret.*"

SPRING, 1937.

### THE EVOLUTION OF A CASUALTY CLEARING STATION ON THE WESTERN FRONT.

The Presidential Address, delivered on 14th October, 1936, at the opening of the Sixty-fourth Session of the Bristol Medico-Chirurgical Society.

BY

RICHARD C. CLARKE, O.B.E., M.B., F.R.C.P.,

*Physician to the Bristol Royal Infirmary.*

In November, 1915, I was posted to No. 19 Casualty Clearing Station. I had been in the 4th Gloucestershire Regiment since the beginning of the war, and had spent from March, 1915, with them in quiet sectors of the line.

No. 19 Casualty Clearing Station was situated then at Doullens, a small market town about 20 kilometres away from the line. The hospital itself was in two buildings about 100 yards apart, one called the Château, which was really the dwelling-house of a

B

cloth mill on the Authie river, and the other a school dignified by the name of the École Moderne, part of which was still used for its original purpose.

The total personnel of the unit was about ninety, with a commanding officer and six officers and a quartermaster. There were six nursing sisters when I joined, but this number was augmented from time to time.

The casualty clearing station had been mobilized about September, 1915, and had arrived in Doullens some time in October. The commanding officer was a regular, who, as second-in-command of a field ambulance, had been taken prisoner at Le Cateau and had then been exchanged. On the whole I liked him very much, but after some twenty years' service in the R.A.M.C. he was a little tied up in red tape, though not half so much as most of his contemporaries.

The rest of the officers were temporary, and as follows: a young general practitioner from South Africa, an Edinburgh graduate of a year's standing, two men just qualified from Belfast and a general practitioner from Derby, who had been a resident in Winchester Hospital; Laurie was his name, and as I shall refer to him a good deal in my narrative, I will describe him further.

Some of you may remember a description of Hinchliffe by his friend Pycroft—"leave him alone with a drum of oil and he'd teach a stolen bicycle to do typewriting"—this would exactly describe Laurie, and in course of time the hospital became full of his devices and inventions, all of which he made himself. Later on he became a most adroit war surgeon, and for operative technique and manual skill I have never seen his equal. When I joined he was engaged in building a large reception hut in the playground of the school, and acquired much merit from the commanding officer and D.M.S. of the army as he had procured all material, wood, tarpaulins, etc., without having signed for it.

Shortly after I arrived some railway trucks containing timber, corrugated iron and other R.E. stores broke loose and piled themselves in the Doullens station. Laurie and I went to see the damage, but he left me suddenly; he was back in no time with our three lorries, which he loaded and re-loaded with all the stuff he wanted, and no one knew that he was not on his lawful occasions.

When I first interviewed the commanding officer I told him that I was by way of being a physician and wanted to do medical work. This quite upset him, and he told me that the policy of the R.A.M.C. was that anyone could do anything and that the medicine and surgery must be equally divided. This rather astonished me, as here was a hospital taking the sick and wounded from a whole division or more, with two hospital trains a week in quiet times for evacuation to the base. This meant, of course, that serious cases had to be kept until they were fit before they were evacuated, and all cases until the arrival of a train. The equipment of the hospital was primitive; for example, all cases were nursed on ordinary army stretchers on trestles in ordinary army blankets. There were six hospital cots, the gift of the British Red Cross Society, which were reserved for officers. This, I remember, was my first "grouse," but the commanding officer insisted that we were a mobile unit. He gradually gave way, however, and we got more beds from the British Red Cross Society for more serious cases, and later on more were issued by the army. At the end of the war we had more than two hundred beds.

The surgery, such as it was, was being done by the general practitioner from South Africa and the Edinburgh graduate. As the consulting surgeon of the army hardly ever appeared—and when he did was as ignorant as we were—the science of war surgery did not get very far in our unit. Looking back on that period in the light of further knowledge, our ignorance was deplorable.

Soon after I joined the casualty clearing station I was introduced for the first time to that dread infection gas gangrene. While I was with the regiment naturally I knew nothing about it, but had noticed that many a man I had evacuated with what was technically called a "Blighty one" had been reported later as "died of wounds," and I never knew till afterwards the reason. At that period we had no idea how it should be dealt with, but later on a special surgery grew up round it—not, however, till the Somme battle two years after the war started.

There were plenty of medical problems at that time: the most urgent was trench fever.

In August, 1915, when I was with the regiment two or three men from one platoon reported sick with pyrexia, headache, limb pains and general malaise, and with typhoid in my mind I visited the rest of that platoon, questioning them, taking their temperatures, and found nine others with the same syndrome. I sent them to the field ambulance, but all were sent back, and I found they were still running evening temperatures. When we came out of the line I went and remonstrated at their return, and reported to the A.D.M.S. that obviously this was an epidemic of some kind and, if not typhoid, a new disease.

He caused a court of inquiry to be held, at which was the sanitary medical officer, a physician in one of the field ambulances and myself. I persuaded these two that it was a new disease, and the findings of the court were "words to that effect," and it was put away and nothing was done—that was my first experience of the disease.

In the casualty clearing station in the winter of 1915–16 the disease was extremely rife and causing an appalling wastage, not of life, because the disease was never fatal, but of personnel.

I well remember an A.D.M.S., an old regular dug-out, coming down to see me at Doullens about it. He came on a Douglas motor-bicycle that he had borrowed from a signaller, and explained rather bitterly that "had he been a —— subaltern of A.S.C. he would have come in a Rolls Royce, but being a poor —— A.D.M.S. he had to borrow a motor-bike." He told me that his general was giving him "socks" owing to the loss of men, and he did not know what to do about it. I was able to explain the clinical aspect of the disease, but did not know then that it was louse-borne, and I could not help him much.

Trench fever was a very curious disease, and very diverse in its manifestations. There were several types of temperature charts, and in some cases the temperature would kick about for months and months: the fever produced a profound debility in time. Some cases, at the start of the disease, had quite definite meningismus, which was always relieved by lumbar puncture, the fluid being normal. Some had intense muscle pain and others bone pains, mostly in the shin, in fact a contemporary Austrian account referred to it as shin-bone fever.

About that time McNee succeeded in transmitting the disease with whole blood,* but his experiments suddenly stopped in 1916, and it was not till the spring of 1918 that the Americans tackled the problem and *proved* it to be louse-borne; but I cannot help feeling that had McNee and a proper research team been put at it in 1916 they would quickly have discovered the etiology, and an enormous wastage of personnel would have been saved by a delousing campaign. I suppose that the cause of the inertia was:—

1. That it was never fatal.
2. That generals and staff did not get it.
3. That very few people realized that trench fever undiagnosed was one of the most potent causes in the loss of morale in individual soldiers and in the production of so-called D.A.H.

There was plenty of war nephritis, which in my opinion was never studied properly. Clinically it was very like puerperal eclampsia, in that massive œdema, convulsions, and rapid recovery of the kidneys were the outstanding features of the disease. I soon discovered that intravenous glucose stopped the fits. After I started giving large quantities of glucose by mouth in all these cases I never saw any more convulsions. I could never explain this phenomenon, nor could anyone else with whom I discussed it, but looking back at it I give you the theory for what it is worth, that hypoglycæmia must have been one of the factors causing the convulsions.

The etiology was never explained, but I am certain that it was a different disease to an ordinary acute nephritis and that cold and damp were not factors,

* "It is almost certain that the body louse must be considered the means of transmitting the disease in nature."—Hunt and McNee, *Quarterly Journal of Medicine*, 1916, vol. ix., p. 442.—[Ed.]

because I saw hospital orderlies and even a nursing sister with it, who were not exposed in any way.

There was a small amount of cerebro-spinal meningitis, and the cases mostly did well with intrathecal injections of anti-serum.

The consulting physician to the 3rd Army was Sir Wilmot Herringham. He had originally been consulting physician to the whole B.E.F., but when it expanded, although he stayed at G.H.Q., he kept on the 3rd Army. From the first he was very friendly and extremely helpful to me, and my association with him continued to the end of the war. He was a very great man of affairs, and with a profound knowledge of everything imaginable. He often stayed with us for a few days, and I never found any subject on which he could not speak with authority. But on the whole I do not think he was a great consulting physician, not being enthusiastic enough in dealing with our particular problems, nor insisting enough on having trained physicians at his casualty clearing stations. He was also a man of extreme prejudices, and I was fortunate in being his friend.

On 1st July the Somme offensive started, and our casualty clearing station was supposed to be in reserve, so that we did nothing on the first day. On the morning of the 2nd I was wakened up at six o'clock and went to the hospital. The commanding officer had previously told the D.M.S., much to my annoyance, that we had accommodation for 800 cases, and when I arrived nearly every square foot of both buildings was filled with lying cases and more were pouring in. I persuaded the commanding officer, after much difficulty, to switch off to the other casualty clearing station in Doullens, though we hadn't then arrived at our complement of 800 cases. As long as I live I shall never forget the next three days—climbing over stretchers, trying to sort the cases out, removing the dead and dying, getting some operated on and sending others to the train, but always finding more cases in unlikely spots.

Never have I felt so utterly helpless and ashamed at being able to do so little. From what I heard afterwards it was the same everywhere. The British Army sustained 90,000 casualties on 1st July, about three times as many as even crude arrangements had been made for, and chaos was a mild word for the result.

Soon after the Somme battle Colonel Gray (now Sir Henry) was appointed consulting surgeon to the 3rd Army, and with all his faults he was, in my opinion, much the best consulting surgeon in the B.E.F., and I think he did more on behalf of the wounded men than any other individual.

In the late autumn of 1916 he brought a visitor, one Dawson Williams, the editor of the *British Medical Journal*, to dine and stay the night. After dinner he produced with great pride two photographs, one of the cerebral vessels and another of the appendix, supposed to have been taken by some new ray process by a Sergeant Shearer, Canadian R.A.M.C. He produced them with a considerable air of mystery. It was after dinner, and Sampson, Laurie, and I asked him who believed in that sort of stuff, and said it was a fraud. Dawson Williams and Gray were terribly offended and the evening went badly after that.

The sequel is amusing. Dawson Williams actually published these photographs in the *British Medical Journal* with a laudatory leading article thereon.

Having got away with that, Shearer got bolder and claimed that he had found a death ray that would kill at a mile. The fraud went on for some time before it was discovered, and is a lesson in what people will believe, especially in war-time.

Soon after Gray joined the 3rd Army we got a real surgical specialist, Sampson, an assistant surgeon from Birmingham. He had had a distinguished academic career, and when the war broke out was already a well-trained young surgeon. He stayed with us till the end of the war and became for judgment, technique and knowledge the very best war surgeon I have ever seen. He was always a glutton for work, and never would rest until there was no more to do.

At the end of 1916 we had orders to move, and built a casualty clearing station in a field at Agnez-lez-Duisans, about four miles west of Arras, in preparation for the spring offensive. The commanding officer, Laurie, Sampson, and myself had many conferences about the lay-out, and eventually we hit on a plan with a very large reception-room, from which a case went through the dressing-room to an arranged destination—such as pre-operation room, the wards, or the evacuation shed. By this method every case had to pass through the dressing-room, and there it was decided whether he should go to the pre-operation room, the sheds for immediate evacuation, the resuscitation ward or to the ordinary ward. From the pre-operation room he went to the theatre, and after operation either straight for evacuation or to one of the wards.

After our Somme experience we were absolutely agreed that side-tracking of cases was to be avoided at all costs.

The day we moved it started to snow, and it froze for the next six weeks. Laurie was in his element at erecting huts and building the hospital generally. He acquired dishonestly a large water tank and quantities of inch pipe. These he laid all over the camp, including a tap in an Armstrong hut I shared with him. He made the roof of our operating hut with a north light running the whole length of it, and many other devices and erections, including an officers' mess of unique design, which turned out to be very uncomfortable.

Eventually we were ready for the offensive at Arras on 9th April. Our rôle in this battle was lying cases, alternate two hundreds, with the casualty clearing station next door, and we had to decide the distribution of our personnel.

Sampson, who had been away operating in a casualty clearing station on the Somme in October, was very insistent that as far as surgery was concerned the success or failure depended on the profitable selection of cases for operation, and we decided that I should do this.

We had by this time acquired another surgeon and Sampson had trained another, so this, with Laurie, gave us four surgeons of our own, and we got two other visiting surgeons from units in quiet areas. This gave us six surgeons, and we got odd officers from attached field ambulances for anæsthetics. This meant that the commanding officer and I were the only officers out of the theatre. The surgeons were to do sixteen-hour shifts with eight hours off, thus giving us four tables going day and night. The commanding officer was to be responsible for the administration and evacuation, and I was responsible for every case until operated on or marked for immediate evacuation.

As a real rush never lasted more than two days I kept on duty continuously, so that one individual was to be responsible for all cases going into the theatre.

In the theatre we had six tables, and the anæsthetist started the next case while the surgeon was dressing the last. Each surgeon had a sister and one trained orderly, and there was a free N.C.O. and a free sister in the theatre to do sterilizing of instruments, etc. When it is realized that by this system no surgeon saw the case he was to operate on until he was under the anæsthetic, the proper selection of cases was of the utmost importance, and that is why we arranged that one man, myself, should stand the racket of any mistake. For this purpose in the dressing-room I had twelve tables to fit stretchers and twelve orderlies, one to each table, and a most efficient corporal in charge. Every case was brought in, put on the table, his wounds undone and a decision made, by me, as to his disposal, either to the resuscitation ward, to the pre-operation ward, to a ward especially kept for the dying, straight to one of the surgical wards or for immediate evacuation.

My problem in a battle was to keep all the surgeons busy with cases really urgently needing operation. At the same time I had not to send so many to the pre-operating room that the last case would have to wait longer for his operation than if sent to the base; and, above all, not to send cases in for operation so badly wounded as to be unlikely to recover. It was a fairly difficult business, but by being kept informed of train times, by interviewing ambulance drivers and finding if more were to come, and being in and out of the pre-operation room and theatre, I was able to get a fair idea of the position.

From the medical point of view the battle of Arras, on 9th April, was far ahead of any battle either before or after, Major J. H. Hartigan (now Director-General Army Medical Services), was A.D.M.S. to the 3rd Army, and made the most efficient arrangements. In our casualty clearing station we were never overwhelmed, and at the end had operated on 30 per cent. of our lying wounded. Our internal arrangements went like clockwork, and for the rest of the war we worked on this plan with very few modifications.

After the battle we were kept fairly busy for three months, but had time to discuss and tackle the various problems of war applicable to a casualty clearing station. All these units had physicians, acting without pay, some of them in the first flight and some of them very much the reverse. Most of them confined their energies to chest wounds, which was a subject fairly easily mastered, and which could not be divorced from other war surgical problems ; so I continued my work of sorting the wounded and preparing cases for operation as well.

Our most vital problem was combating shock and hæmorrhage, and as almost every case was heavily infected, time was the vital factor. The speed at which a case became heavily infected was appalling, and in our Doullens days we had grown hæmolytic streptococcus from the heart blood of a man wounded eighteen hours before. Morphia and warmth we could give, but not time, and the task of getting a shocked case ready for operation before he was hopelessly infected was the problem which confronted us.

There is not time to go through all our experiments and mental processes, but in our casualty clearing station we came to very definite conclusions :—

1. That morphia was hard to overdo.

2. Heat was essential, and for this purpose Laurie devised a stretcher cage covered with blankets in which we put a paraffin lamp. It seemed very dangerous, but curiously enough we had no accident of any kind, and it answered admirably. It became known as "the cooker."

3. We did not like restoratives before operation, as it often lulled the surgeon with false security into doing more than the case would stand.

4. That blood transfusion was marvellous for hæmorrhage, but very disappointing for shock.

It was, of course, very difficult to distinguish between shock and hæmorrhage; but in war-time the answer to the question, "Where is the blood in a shocked case ?" is often "On the battlefield." Regimental experience had shown me how much oozing went on from ordinary flesh wounds, and how frequently one had to change dressings of cases that had to be kept in a regimental aid post for some hours, and one was apt to forget how much blood a man had lost when one saw him in a casualty clearing station with dry wounds.

Directly after the battle of Arras we started on blood transfusion, then in its infancy. Our first experiment was artery to vein direct. The first case was done by Laurie, and we were so interested, and went on for so long, that the donor, a florid type of man, became the colour of the recipient, and the recipient, quite bled out from a torn femoral artery, became absolutely florid. In the light of future experience I think four pints was a conservative estimate. We tried this method again a few times, but never made it go so well.

After much trial and error we eventually adopted a method, mostly devised by Laurie, by which a man received five pints of mixed fluid, three of hypertonic saline and two of blood; but even then, although the effect on the patients was wonderful, it was a method unsuitable for those requiring a long operation, and the ideal type of case was a man with a shattered leg needing a rapid amputation under gas and oxygen.

Blood transfusion was also unsuitable for rushes, as it took too much time.

In July, after much agitation, we got a portable X-ray apparatus, and we were lucky enough to get Finzi, from St. Bartholomew's, to work it, and in a short time it became indispensable.

Later in 1917 nursing sisters were trained to give anæsthetics, and it turned out to be a most successful experiment. Before we had to rely mostly on attached officers from the ambulance during rushes, and whether it was from lack of practice or lack of training, I am bound to admit most of them were rather bad. I have even seen artificial respiration being performed at all four tables at the same time. In fact, it became a standing joke in our unit that when a "brass hat" or other big-wig came someone was always restoring a case from an overdose. We only actually had two deaths from anæsthetics, but when the nursing sisters came there was never any more trouble, and they even became experts with gas and oxygen.

In June, 1917, our commanding officer left us to go to a division, and Colonel Hartigan came from the 3rd Army to command us. He was a great loss to the 3rd Army, as he was a most efficient staff officer, and the staff work of the 3rd Army was never the same again. He left us in the November and was replaced by R. C. Dunn, a Liverpool surgeon and a Territorial, who stayed with us till the end of the war. After he came everything went with a swing, and during his command our unit was a very pleasant one.

We stayed at Agnez-lez-Duisans throughout the winter till the March offensive. At first we were not involved, but on the 28th Arras and Vimy Ridge were attacked, and just as we were getting busy we received orders to retreat. I understand that the exact time and place of the attack was known to our intelligence staffs some five days before, and why one of the casualty clearing stations in the group was not moved back and opened ready to receive the wounded will ever remain a mystery. We were all very angry at the time, and the maddening thing about it was that the attack was unsuccessful and we need never have moved at all. The other casualty clearing station at Duisans was overwhelmed and unable to deal with the situation properly.

I myself took some of the personnel and opened a place for walking wounded in some French huts at Haute Avesnes, some six miles back, and for four weeks had very little to do before rejoining the unit which had opened at Frevent in tents.

My chief memory of my time at Haute Avesnes was a visit from an A.D.M.S. of a division—a regular of the old type. He got very heated, and said that he had always said it was unsafe to have an X-ray plant at a casualty clearing station, and now he was right, as No. — Casualty Clearing Station had lost their plant in the retreat. I pointed out to him that the X-ray plant had been working for nearly a year and must have saved many lives and limbs. I also could not refrain from saying that the British Army had lost a good many guns of greater value. This only made him angry, and he left in high dudgeon.

This incident I give as being typical of the outlook of some of the senior officers of the R.A.M.C.

Soon after I got to Frevent the first wave of the 1918 influenza epidemic started. It was a genuine pandemic and, things being quiet on our front, I was able to study the disease. The incidence, I calculated, was something over 80 per cent. I took a lot of trouble to obtain this figure, and if anything it was an underestimate. It was a three-day disease with very few catarrhal complications, and the pneumonic incidence was practically nil. Nevertheless, my own private view is that it came to the rescue of the allied armies. If we take the dates of the German offensives, all of which were more or less staged by March; the first was on 21st March, the next on 29th March at Arras, the next on 9th April in Flanders, and the next on 14th May on the Aisne, soon after which this pandemic started in both armies.

This must have hung up the German army considerably, and they were not ready till 16th July for their next offensive, by which time our defences were organized and enough Americans had arrived in France to hold them up and eventually to turn the tide.

Oddly enough, in a fairly extensive reading of war literature, Ludendorff is the only writer I have read who has mentioned this fact as one of the causes of the German delay and eventual debacle.

On 21st August the 3rd Army attacked, and the higher command succeeded in keeping it secret, as they did the attack of the 4th Army on 8th August. I might mention here that all other offensives had been advertised for weeks before, and the date and place was common knowledge in the army.

The disadvantage from our point of view of this secrecy was that we were caught on the hop and only heard of it the night before, so we had no visiting teams at the beginning of the attack.

We had at this time five surgeons of our own, three of them first class, and we managed very well. Fortunately the enemy used mustard gas, and gas casualties were an easy problem compared with those of high explosives and machine-guns. I had also discovered by this time that the vast majority of chest cases with either small or no foreign bodies retained could be evacuated forthwith, and later on I received statistics from the base which told me that seventy-nine out of eighty-two cases I sent down during this battle had been evacuated to England. This was a good save on our accommodation.

This battle was such a success that we were soon left miles behind the line and moved up to a siding at Boisleau, south of Arras, opening again with tents ready for the attack on the Hindenburg line on 25th September. This attack was an amazing success, and we were able to deal with our casualties efficiently, and again we were left behind by the rapidly advancing British Army. Two of our surgeons went off on operating teams, and I took a section of the unit and opened a walking wounded station on the Bapaume-Cambrai road which was open for the next attack. I hadn't much to do myself and saw the work of the other three casualty clearing stations. Their organization was not in any way as good as ours.

Eventually the whole unit moved to Caudry, a small manufacturing town east of Cambrai, and opened in a large lace factory which had been bereft of its machinery and used by the Germans as a hospital. Being in a building our lay-out was a bit difficult, but we were able to arrange our " continuous flow " system. Here we took the wounded in the last two offensives in the war, in which the casualties were fairly heavy.

By this time I think I had mastered the task of sorting the wounded. Looking back on my life, I think that I did this better eventually than anything I have ever done before or since. I had got to know how much a wounded man could stand in the way of operation and the necessity of operation in every individual case. In the last battle, Laurie having told me that he wanted to do abdominal cases, in two days I selected him twenty-one cases, all of which survived, a record we were rather proud of at the time.

While we were at Caudry we were greatly bothered by the absence of hospital trains, as the railway line was continually being blown up by delayed mines. In Caudry itself there was a railway bridge crossing a main road quite intact. None of us liked to look at this at all, and I remember how we used to hurry past. It was said that the bridge had been thoroughly examined by the R.E. and pronounced safe. A few days before the Armistice at nine o'clock in the morning up it went, and four of our sisters had a narrow escape while going to their billets off night duty.

Just before the Armistice the second wave of influenza started, nothing like the pandemic in May or June, but a most dreadful disease—a " black death." We were getting more deaths daily in our unit than we got in battles. I kept a special ward of forty beds for the pneumonia cases well staffed by nursing sisters, and although I was admitting cases to it continually I never filled it up, so great was the mortality. Quite a common occurrence was to receive an ambulance with eight sitting cases, half of which were moribund with no chance at all.

While I had no time for any scientific observations, I got the impression that the mortality of those showing consolidation of the lungs was 100 per cent.

It was for me by far the worst time in the war, and took away all pleasure from the Armistice. I have never felt so helpless, as no treatment seemed of any avail. I did, however, arrive at one definite conclusion, namely that for influenza a journey of sixty or eighty kilometres in an ambulance was the very worst thing that could happen, and, Herringham having gone home and left me on duty for him, I was able to get a circular sent to all the units in the 3rd Army to send all influenza cases down as lying cases. I like to think that this helped matters.

I was demobilized at the end of January, 1919, when Herringham came back.

To sum up, I think I had as interesting a war as anyone in our profession, as I had seen a casualty clearing station develop from a really crude and hopelessly inefficient unit to one capable of dealing with large numbers of casualties in an efficient manner. Admittedly the process was slow, at times exasperatingly slow. At the time I suppose one was too apt to be critical of the regular R.A.M.C., and looking back on it in a dispassionate manner, the blame must be given, not to the R.A.M.C., but to successive Governments, who provided the Army in peace time with a medical service "on the cheap." The effect of this on most of the senior officers, hampered all their service lives by finance, was exactly what one would expect. They became hide-bound and tied up in red tape and economy, no doubt a very desirable asset in peace time, but unsuitable in a war of the magnitude of the last one, and it took some time for them to adopt a different mental outlook. There were, of course, brilliant exceptions—Hartigan, for instance—and looking back on those times, it was wonderful that so much was accomplished in the end.

